# The main influencing factors of customer satisfaction and loyalty in city express delivery

**DOI:** 10.3389/fpsyg.2022.1044032

**Published:** 2022-10-24

**Authors:** Zheng Lei, Huawei Duan, Liping Zhang, Daji Ergu, Fangyao Liu

**Affiliations:** ^1^School of Management, Xihua University, Chengdu, Sichuan, China; ^2^School of Electronic and Information, Southwest Minzu University, Chengdu, China

**Keywords:** city express, quality of service, customer satisfaction, customer loyalty, FAHP, stratification regression analysis

## Abstract

At present, customers’ low satisfaction and loyalty to city express service have restricted the development of city express. It is particularly important to analyze the factors causing customers’ low satisfaction and loyalty, which will promote the development of city express industry effectively. Based on SERVQUAL model and CCSI model, this paper constructs a new evaluation index system from the perspective of service quality. Through this new system, this paper first explores the factors that affect customers’ satisfaction and loyalty, respectively, by fuzzy analytic hierarchy process (AHP) and hierarchical regression analysis, taking the expected and perceived service quality as conversion variables. And then it analyzes the common factors that affect customers’ satisfaction and loyalty comprehensively. These two analyses will provide reference for solving the problem of low customer satisfaction and loyalty of city express enterprises. The results show that popularity and credibility, delivery time commitment, and mailing security are the common main factors affecting customer satisfaction and loyalty. Easy-to-understand receipts, the three-level index corresponding to the empathy dimension, is the most significant factor affecting customers’ loyalty in city express industry; Delivery time commitment, the three-level index corresponding to the reliability dimension, is the most significant factor affecting customers’ loyalty in city express industry.

## Introduction

City express service, also known as “the last mile logistics,” is one of the three major services in the express industry, with a large market demand and plenty of room for growth. According to State Post Bureau data, the volume of express delivery business in the same city has increased from 2.29 billion pieces in 2013 to 12.17 billion pieces in 2020. Despite the epidemic, it is still growing steadily, with a year-on-year growth of 10.2%, accounting for 14.6% of total express delivery business, making it the fastest growing sub-industry in China’s logistics industry. The mature development of city express has become a strong guarantee for the improvement of the operational efficiency of the urban economy.

However, despite the business’s growth, service offerings, and product diversification, extensive operation mode has been unable to satisfy demand from customers. The city express industry in China has entered a phase of transition from the initial “labor-intensive” distribution of commodities reliant on manual labor and antiquated mechanical facilities to the “intelligent logistics” of high-tech transformation, artificial intelligence, big data, and high-tech. As a result, issues such a lack of specialization, excessive distribution costs, and subpar after-sales support grow more and more prevalent, which lowers customer happiness and loyalty. The “throwing express” event involving Sto has received a lot of media attention. Only in the first half of 2021, the net loss reached 144 million yuan, and the earnings declined 285.69% year on year, J&T ranked last in the China Post’s service satisfaction survey for the third quarter of 2021 at the end of 2021. Many phenomena show that enterprises should no longer simply consider the cost and price, but should pay more attention to customer satisfaction, and change the pursuit of single profit maximization into overall profit maximization. Therefore, how to improve customer satisfaction and loyalty to occupy more market share has become a difficult problem plaguing the development of enterprises.

In the service industry, the overall service quality of enterprises determines the level of customer satisfaction and loyalty. Meanwhile, it is the key to increasing the market share of enterprises. Existing studies on the influencing factors of satisfaction and loyalty mainly focus on hotels and restaurants, e-commerce, tourism, supermarkets, banks, and other aspects. However, there are few studies on customer satisfaction of city express delivery. Therefore, this paper draws on the experience of other industries in customer satisfaction to study city express. The selected research methods also draw on the research contents of other industries. As a result, the motivation for this paper is to determine which aspect of service improvement can more effectively and quickly improve customer satisfaction of city express delivery and thus increase customer loyalty. The main factors affecting customer satisfaction and loyalty are explored by comparing the factor differences between customers’ expected revenue before purchasing service and actual revenue after receiving service.

## Literature review

Compared with other industries, the products provided by service industries are services. Different from tangible products, service products are more difficult to be measured and perceived due to their unique intangibility, inseparability, heterogeneity, and perishable nature.

The research content of this paper mainly involves three aspects: service quality, customer satisfaction, and customer loyalty. The currently recognized service quality evaluation model was that proposed by Zeithaml et al. (1985). This model measures service quality by measuring the difference between customer service expectation and service perception from five aspects: reliability, responsiveness, security, empathy, and tangible. In addition, Oliver’s “expectation inconsistency theory” laid a solid foundation for the research of satisfaction. The research on customer satisfaction in China began in the early 21st century. The research on the satisfaction of express delivery industry focuses on the analysis of influencing factors and innovative evaluation methods. The “attitude loyalty theory” proposed by Hallowell in 1996 provides a theoretical basis for indirect evaluation of service quality by using customer loyalty. The following is an overview of the correlation among the three:

In the field of express logistics, researches on the relationship between service quality and customer satisfaction show that service quality is the primary factor affecting customer satisfaction. In 1980, Oliver (1980) pointed out that service quality is an important driver of customer satisfaction, whether it is transaction-oriented satisfaction or cumulative satisfaction. Based on the theoretical basis of customer satisfaction and express service, Qiyuan (2022) found that service quality has a significant impact on customer satisfaction, and perceived quality is the main factor. However, through systematic theoretical analysis and practical investigation, he found that the corporate image, service quality, and service price had a relatively significant impact on customer satisfaction. Among them, corporate image and service price are the basic factors affecting customer service reliability, responsiveness, convenience, and guarantee were low, it would lead to low customer satisfaction and affect the overall level and quality of express service.

On the whole, there is a significant positive correlation between customer satisfaction and loyalty. As early as 1993, Oliver (1993) proposed that there was a non-linear relationship between customer satisfaction and loyalty. Ming and Luming (2015) empirically analyzed the factors affecting customer loyalty and finally determined that customer expected quality, customer perceived quality, customer perceived value, corporate image, and customer satisfaction were the most important factors among many factors. Zhiyan et al. (2021) interpreted the perceived value with the perceived service quality and the perceived service cost, and established a structural equation model based on the CCSI model. Finally, he proved that perceived service quality and perceived service cost had the most significant impact on customer satisfaction of logistics distribution service, and the impact of brand image could also not be ignored. Meanwhile, customer satisfaction significantly determines customer loyalty.

At present, there are few studies on the relationship between express logistics service quality and customer loyalty. By integrating existing domestic and foreign literature, scholars generally believe that there is a positive correlation, but no direct influence. Rao et al. (2011) found that logistics distribution cost and service quality are key factors affecting customer loyalty in their research. In the study of Taylor and Baker (1994), there was no strong positive correlation between service quality and repurchase tendency. Taylor and Xiao (2010) pointed out that the main factor affecting purchase intention is customer satisfaction rather than service quality, and the customer satisfaction has a significant influence on purchase intention, which is greater than that of service quality. With customer satisfaction and customer trust as the intermediary variable, Ying et al. (2016) research the relationship between logistics service quality and customer loyalty. They found that emergency handling quality, information interaction, and logistics distribution quality had a direct and positive impact on customer loyalty. And the quality of personnel service indirectly affects customer loyalty by affecting the intermediary variable of customer trust.

## Research framework and hypothesis development

In order to explore the main factors affecting customer satisfaction and loyalty of same-city express delivery, and at the same time make the results more consistent with the status quo of China’s same-city express delivery industry, the CCSI model is combined to correct the model based on the SERVQUAL model in this paper.

**Figure 1:**
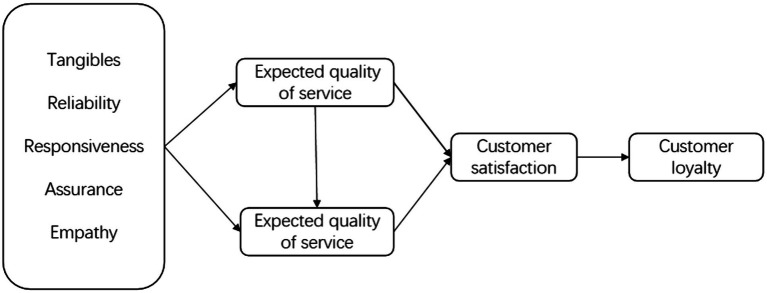
Evaluation model.

In this paper, expected service quality and perceived service quality are added as conversion variables between service quality and customer satisfaction. And the value of expected service quality minus perceived service quality is used as a method to evaluate the influencing factors of customer satisfaction and loyalty. The structural model is shown in Figure 1.

The components of the structural model in this paper present a causal relationship. This paper analyzes the expected service quality of customers before enjoying the service and the actual perceived service quality after experiencing the service from the five dimensions of tangibility, reliability, responsiveness, assurance, and empathy of the express service in the same city. Customers’ subjective requirements for perceived service quality will be higher as expected service quality increases. When the expected service quality is high and the perceived service quality is low, the difference between perceived service quality and expected service quality will be smaller, and the customer satisfaction will be low. The decrease of customer satisfaction will lead to the decrease of customers’ trust and loyalty to the product, and they will increase conversely. In a word, the model takes into account the comprehensive satisfaction of customers when they choose services and after they experience services, so as to evaluate customers’ future purchasing attitudes and achieve synchronous improvement of customer satisfaction and loyalty.

Based on the existing research of city express and combined with the structural model, the author proposed the following hypotheses:

Shuai (2015) put forward that the delivery quality of goods has the greatest impact on customer satisfaction. Customers are most concerned about the security of deliverables, followed by the timeliness and timeliness of delivery.

*H1*: Timeliness and security factors under the reliability dimension are the most influential factors that affect the change degree of satisfaction of city express delivery.

Zhiyan et al. (2021) solved the problem and concluded that in the logistics distribution industry, expected service quality has a negative direct impact on customer loyalty, while perceived service quality has a positive direct impact on customer loyalty.

*H2*: The expected service quality of city express is negatively correlated with customer loyalty.

*H3*: The perceived service quality of city express is positively correlated with customer loyalty.

Xuefang et al. (2015) pointed out that in the banking industry, customer satisfaction is not equal to customer loyalty, but customer satisfaction has a significant positive correlation with customer loyalty, and the most significant factor affecting customer satisfaction is also the most prominent factor affecting customer loyalty.

*H4*: The most significant factor for improving the satisfaction of city express delivery is also the most effective factor for improving customer loyalty of city express delivery.

## Research design

### Evaluation system construction

In this paper, the implicit variables and explicit variables in CCSI model and SERVQUAL model are integrated, and the main reasons for the slowdown of the development of China’s urban express delivery industry and the factors causing this phenomenon are combined with the special national conditions of China in the report on the Market Operation management and Investment Prospects of China’s Urban Express Delivery Industry in 2021–2027. Establish an index system suitable for the express industry in the same city. This index system is divided into three levels, as shown in Table 1.

**Table 1:** Satisfaction indication system.

First-level indicators	Second-level indicators	Third-level indicators
Customer Satisfaction	Tangibles	Equipment
		Courier staff’s dress
		The inquiry way of order
		The complaint channels
		Complaint channels
	Reliability	Popularity and credibility
		Delivery time commitment
		Mailing security
		Matching degree between price and service
	Responsiveness	Diversified distribution methods
		Coverage of outlets
		Order processing speed
		Problem processing speed
	Assurance	Delivery person meter
		Shipment tracking method
		Staff professional ability
		Staff service attitude
	Empathy	Exclusive APP for business handling
		Humanized way of receiving
		Gradient price standard
		Easy-to-understand receipts

### Questionnaire and data collection

This chapter designs the questionnaire according to the index system. The collected data will be used as the basic data of hierarchical analysis and fuzzy comprehensive analysis.

The questionnaire is divided into three parts, namely, “basic information,” “survey on the perceived service quality of city express,” and “survey on the expected service quality of city express.” Basic information mainly includes “have you ever used the city express?,” “frequency of use,” “age,” “income,” “the company which frequently choose,” the reason for choosing this company’s services for a long time.” According to the satisfaction index system, the survey of perceived service quality (expected service quality) of city express delivery is divided into 5 parts, with a total of 25 questions and measured by Likert-type scale.

This questionnaire was collected from July 2021 to August 2021. It was distributed on multiple network platforms and offline, as well as on WeChat, Weibo, and other communication apps. A total of 327 questionnaires were collected, excluding 20 invalid ones, 307 valid ones were obtained with an effective recovery rate of 93.88%. Among the 307 valid questionnaires, 45 did not use city express, accounting for 14.66%; 32.57% used once a week; Use it once or twice a week 33.55%; Three to five times a week 17.26%; More than 6 times a week 1.95%.

Combined with the questionnaire data, the basic characteristics of the sample are as follows:

1. In terms of age, the main customers of city express are 18–40 years old, accounting for 82.44%;
2. In terms of income, customers with an income of 2,000–8,000 yuan use city express the most, accounting for 75.19%;
3. Yunda Express (23.66%), SF Express (22.9%), and ZTO Express (15.27%) are the top three companies that often choose to provide local express services.

### Reliability and validity analysis

In order to ensure that the questionnaire can accurately reflect the question, the reliability and validity of the questionnaire are analyzed first. In the reliability analysis, Cronbach’s coefficient (Cronbach α) was used to measure the reliability of questionnaire data. In the validity analysis, KMO sampling suitability quantity and Bartlett Test of Sphericity were used to analyze whether there was a strong correlation between each measurement index. The specific results are shown in Tables 2, 3. The reliability coefficients of the questionnaire are all greater than 0.9, and the KMO value is greater than 0.8. Moreover, the Bartlett sphericity test value of *p* is 0.000, and the variables are not independent.

**Table 2:** Reliability.

	Expected quality of service	Perceived quality of service
Cronbach’s Alpha	0.950	0.938
Cronbach’s Alpha	0.950	0.939
Number	25	25

**Table 3:** Validity.

		Expected quality of service	Perceived quality of service
KMO		0.960	0.950
Bartlett test	Approx. Chi-Square	3235.411	2781.209
	df	300	300

## Findings

### Main influencing factors of customer satisfaction

In order to get the main factors that affect the satisfaction of local express delivery, this paper chooses fuzzy analytic hierarchy process to explore.

First, according to the expert questionnaire, using analytic hierarchy process to get the weight, and normalized processing. The calculation results show that the weight vector of second-level index relative to first-level index is Customer satisfaction = (0.12, 0.47, 0.10, 0.23, 0.08); The weight vector of the third-level index relative to the second-level index is: Tangibility = (0.10, 0.05, 0.31, 0.17, 0.37), Reliability = (0.12, 0.42, 0.23, 0.23), Responsiveness = (0.35, 0.35, 0.16, 0.14), Assurance = (0.07, 0.25, 0.51, 0.17), and Empathy = (0.18, 0.35, 0.39, 0.08).

Secondly, score set V = (very dissatisfied, dissatisfied, general, satisfied, and very satisfied) is defined to set the corresponding scoring range, and S is the corresponding score value of comments in V, as shown in Table 4.

**Table 4:** Questionnaire score table.

Comment	Very dissatisfied	Dissatisfied	General	Satisfied	Very satisfied
Score (S)	0–40	40–60	60–80	80–90	90–100

Then, fuzzy evaluation matrices of expected service quality and perceived service quality were established respectively, and the comprehensive scores of secondary indicators were shown in Table 5.

**Table 5:** Second-level index score.

Second-level indicator	Composite scores	Tangibility	Reliability	Responsiveness	Assurance	Empathy
Expected quality of service	89.9	89.9	90.3	90.1	88	90.1
Perceived quality of service	89.3	89.9	89.7	91	88.9	89.7
Difference value	−0.6	0.00	−0.6	0.9	0.9	−0.5

As can be seen from Table 5, the difference between reliability and empathy is−0.6 and-0.5, indicating that the perceived value of customers’ actual experience does not meet customers’ expectations. Improving reliability and empathy can effectively improve overall customer satisfaction. Then, the fuzzy comprehensive evaluation method was used to score the reliability of the second-level indicators and the third-level indicators corresponding to empathy, respectively. The results are shown in Tables 6, 7.

**Table 6:** Reliability corresponds to the third-level index scores.

Third-level indicator	Expected service quality score	Perceived service quality score	Difference value
Popularity and credibility	89.9	88.1	−1.8
Delivery time commitment	92.2	90	−2.2
Mailing security	91.9	89.5	−2.4
Matching degree between price and service	88.5	89.5	1

**Table 7:** Empathy corresponds to the three-level index score.

Third-level indicator	Expected service quality score	Perceived service quality score	Difference value
Exclusive APP for business handling	90.3	88.4	−1.9
Humanized way of receiving	89.8	89.1	−0.7
Gradient price standard	88.5	89.2	0.7
Easy-to-understand receipts	92.2	89.6	−2.6

It can be seen from Table 6 that the third-level index corresponding to the second-level index “Reliability” has the largest difference value of “Mailing security” with a different value of-2.4, followed by “Delivery time commitment” and “Popularity and credibility” with a different value of-2.2 and-1.8, respectively. In terms of “Matching degree between price and service,” customer perception value higher than expected. As can be seen from Table 7, the third-level indicator corresponding to the second-level indicator “Empathy” has the largest different value of “Easy-to-understand receipts” with a difference value of-2.6, followed by “Exclusive APP for business handling” and “Humanized way of receiving” with a different value of-1.9 and-0.7, respectively. In terms of “Gradient price standard,” customer perception value is slightly higher than expected. Therefore, the main factors affecting customer satisfaction of express delivery in the same city are “Popularity and credibility,” “Delivery time commitment,” “Mailing security,” “Exclusive APP for business handling,” “Humanized way of receiving” and “Easy-to-understand receipts.” Among them, the difference value of “Easy-to-understand receipts” provided is −2.6, which is the most significant factor to improve customer satisfaction.

### Main influencing factors of customer loyalty

According to the existing literature, it cannot be directly proved that there is an inevitable connection between the five dimensions of service quality and customer loyalty in the city express delivery industry. In order to solve this problem, hierarchical regression method is adopted in this paper. It is known that there is a positive correlation between customer satisfaction and loyalty. The research in 5.1 proves that perceived service quality and expected service quality have a significant impact on customer satisfaction. Similarly, the five dimensions of service quality have a significant impact on perceived service quality and expected service quality. Therefore, the analysis method in this paper is divided into three layers. The specific results are shown in Table 8.

**Table 8:** Results of stratified regression analysis.

		Tier 1: Customer satisfaction→Customer loyalty					Tier 2: Add service quality (expectations/perceptions)					Tier 3: Add five dimensions of service quality (expectation/perception)				
		B	SE	*t*	*p*	β	B	SE	*t*	*p*	β	B	SE	*t*	*p*	β
Customer Satisfaction		0.447	0.079	5.649	0.000	0.331	−0.488	0.162	−3.013	0.003	−0.360	−0.342	0.107	−3.198	0.002	−0.253
Expected quality of service							−0.841	0.133	−6.333	0.000	−1.036	−0.152	0.108	−1.411	0.159	−0.187
Perceived quality of service							0.852	0.131	6.522	0.000	0.961	0.143	0.095	1.513	0.132	0.162
Expected quality of service	Tangibles											−0.275	0.054	−5.144	0.000	−0.341
	Reliability											−0.393	0.042	−9.248	0.000	−0.517
	Responsiveness											−0.108	0.043	−2.522	0.012	−0.141
	Assurance											−0.167	0.045	−3.742	0.000	−0.224
	Empathy											−0.128	0.046	−2.770	0.006	−0.165
Perceived quality of service	Tangibles											0.269	0.055	4.881	0.000	0.312
	Reliability											0.455	0.051	9.010	0.000	0.538
	Responsiveness											0.056	0.047	1.179	0.240	0.065
	Assurance											0.202	0.047	4.308	0.000	0.249
	Empathy											0.155	0.047	3.276	0.001	0.188
R^2^		0.109					0.237					0.714				
Adjusted R^2^		0.106					0.228					0.699				
*F*		*F* (1,260) =31.916, *p* = 0.000					*F* (3,258) = 26.703, *p* = 0.000					*F* (13,248) = 47.551, *p* = 0.000				
△R^2^		0.109					0.128					0.477				
△F		*F* (1,260) = 31.916, *p* = 0.000					*F* (2,258) = 21.572, *p* = 0.000					*F* (10,248) = 41.293, *p* = 0.000				

The basic data used to analyze the main influencing factors of customer loyalty comes from the loyalty questionnaire. According to the evaluation model and literature reference, the index system for evaluating customer loyalty is basically the same as customer satisfaction. It also makes it easier to compare satisfaction with loyalty.

As can be seen from Table 8, tier 1 takes customer satisfaction as the independent variable and customer loyalty as the dependent variable for linear regression analysis. As can be seen from the above table, the model R square value is 0.109, meaning that customer satisfaction can explain 10.9% of the reasons for the change of customer loyalty. The model passed the *F*-test (*F* = 31.916, *p* < 0.05), that is, customer satisfaction must have an impact on customer loyalty, and the model formula is: customer loyalty = 0.030 + 0.447* customer satisfaction. The final specific analysis shows that: the regression coefficient value of customer satisfaction is 0.447, and presents a significant value (*t* = 5.649, *p* = 0.000 < 0.01), which means that customer satisfaction has a significant positive impact on customer loyalty.

When expected service quality is added on the basis of tier 1, *F* value changes significantly (*p* < 0.05), which means expected service quality, and perceived service quality has explanatory significance to the model. In addition, R square value increased from 0.109 to 0.237, indicating that expected service quality, perceived service quality can have 12.8% explanatory power to customer loyalty. Specifically, the regression coefficient value of expected service quality was −0.841 and presented a significant value (*t* = −6.333, *p* = 0.000 < 0.01), which means that expected service quality has a significant negative impact on customer loyalty. The regression coefficient of perceived service quality was 0.852 and presented a significant value (*t* = 6.522, *p* = 0.000 < 0.01), indicating that perceived service quality has a significant positive impact on customer loyalty.

After adding five dimensions of service quality on the basis of tier 2, the change of F value presents significant (*p* < 0.05), which means that the addition of five dimensions has explanatory significance to the model. In addition, the value of R square increases from 0.237 to 0.714, which means that the five dimensions of perceived service quality and expected service quality can explain 47.7% of customer loyalty. Specifically, it can be seen that the five dimensions of expected service quality have a negative impact on customer satisfaction, while the five dimensions of perceived service quality have a positive impact on customer loyalty except responsiveness.

According to the regression coefficient, factors with a regression coefficient higher than 0.3 can be taken as the main factors affecting customer loyalty, and it can be concluded that the main factors affecting customer loyalty are reliability of expected service quality and reliability of perceived service quality. Again, the corresponding three-level indicators are taken as independent variables, and the reliability of expected service quality and perceived service quality is taken as dependent variables for regression analysis. The results are shown in Tables 9, 10.

**Table 9:** Regression results of three-level indicators and expected service quality reliability.

	Unstandardized coefficients		Standardized coefficients	*t*	*p*	VIF	R^2^	Adjust R^2^	*F*
	B	SE	Beta						
Popularity and credibility	0.278	0.025	0.314	11.257	0.000	1.386	0.856	0.854	*F*(4,257) = 381.286, p = 0.000
Delivery time commitment	0.279	0.024	0.339	11.478	0.000	1.553			
Mailing security	0.246	0.029	0.266	8.412	0.000	1.785			
Matching degree between price and service	0.271	0.029	0.282	9.372	0.000	1.615			
D-W	2.091								

**Table 10:** Regression results of three-level indicators and perceived service quality reliability.

	Unstandardized coefficients		Standardized coefficients	*t*	*p*	VIF	R^2^	Adjust R^2^	*F*
	B	SE	Beta						
Popularity and credibility	0.211	0.027	0.257	7.965	0.000	1.554	0.828	0.825	F(4,257) = 308.735, p = 0.000
Delivery time commitment	0.294	0.023	0.379	12.647	0.000	1.339			
Mailing security	0.223	0.029	0.244	7.813	0.000	1.450			
Matching degree between price and service	0.286	0.027	0.326	10.700	0.000	1.385			
D-W	2.206								

## Results and conclusions

Through the above analysis, it can be concluded that the main factors affecting customer satisfaction of city express delivery are in sequence: “Easy-to-understand receipts,” “Mailing security,” “Delivery time commitment,” “Exclusive APP for business handling,” “Popularity and credibility,” and “Humanized way of receiving.” The main factors affecting customer loyalty are in proper order: “Delivery time commitment,” “Matching degree between price and service,” “Popularity and credibility,” and “Mailing security.” Thus, the results of the previous research hypothesis can be obtained, as shown in Table 11.

**Table 11:** Experimental results of the hypothesis.

Hypothesis	Inspection results
H1: Timeliness and security factors under the reliability dimension are the most influential factors that affect the change degree of satisfaction of city express delivery.	Nonsupport
H2: The expected service quality of city express is negatively correlated with customer loyalty.	Support
H3: The perceived service quality of city express is positively correlated with customer loyalty.	support
H4: The most significant factor for improving the satisfaction of city express delivery is also the most effective factor for improving customer loyalty of city express delivery.	Nonsupport

There are various reasons why the test results do not conform to the hypothesis. According to the results of influencing factors, we can know that, compared with the research results of the main influencing factors of customer satisfaction in other industries, “Easy-to-understand receipts” is the most important factor to improve customer satisfaction of city express delivery. The main reason is that the main delivery items of city express delivery are documents and other small parcels. The main service objects are enterprises, companies, schools, government agencies, etc. Simple and clear documents can be used for account reimbursement and information storage more quickly, saving users’ time.

By comparing the main influencing factors of customer satisfaction and loyalty, it can be found that “Popularity and credibility,” “Delivery time commitment,”

“Mailing security” are common factors affecting customer satisfaction and loyalty, but the importance of these three factors for satisfaction and loyalty is different. The reason for this result is that satisfaction is the evaluation of the service purchased in a single time or a short time, while loyalty is the long-term perception of the enterprise and its services.

The research methods adopted in this paper mainly refer to the satisfaction research methods of other industries. The purpose of this paper is to provide a reference for the research of urban express satisfaction. City express enterprises should combine the actual situation when choosing how to improve customer satisfaction quickly.

In the future, the author’s research content will be divided into two aspects. The first is to research the methods to analyze the customer satisfaction of express delivery in the same city. The second is the research on the evaluation system and index of customer satisfaction of city express. In order to expect for the city express industry to put forward more development proposals.

## Data availability statement

The original contributions presented in the study are included in the article/supplementary material, further inquiries can be directed to the corresponding author.

## Author contributions

ZL: methodology, data analysis, and writing-original draft preparation. HD: conceptualization, writing-review and editing, and funding acquisition. LZ: index system and questionnaire design. DE: software and validation. FL: data collection and software. All authors contributed to the article and approved the submitted version.

## Funding

This work was supported by Social Science Planning project of Sichuan Province (grant number SC21B116); Natural Science Foundation Project of Sichuan Province (grant number: 2022NSFSC1865); and Key scientific research fund of Xihua University (grant number: Z17131).

## Conflict of interest

The authors declare that the research was conducted in the absence of any commercial or financial relationships that could be construed as a potential conflict of interest.

## Publisher’s note

All claims expressed in this article are solely those of the authors and do not necessarily represent those of their affiliated organizations, or those of the publisher, the editors and the reviewers. Any product that may be evaluated in this article, or claim that may be made by its manufacturer, is not guaranteed or endorsed by the publisher.
